# A Study on the Association Between Myopia and Elevated Intraocular Pressure Conducted at a Tertiary Care Teaching Hospital in Gujarat, India

**DOI:** 10.7759/cureus.28128

**Published:** 2022-08-18

**Authors:** Ashka Patel, Darshvi Patel, Vaishali Prajapati, Manoj S Patil, Deepika Singhal

**Affiliations:** 1 Ophthalmology, Gujarat Medical Education and Research Society (GMERS) Medical College and Civil Hospital, Ahmedabad, IND; 2 Research and Development, Jawaharlal Nehru Medical College, Datta Meghe Institute of Medical Sciences, Wardha, IND

**Keywords:** optic neuropathy, glaucoma, goldmann applanation tonometer, intraocular pressure, myopia

## Abstract

Introduction

Glaucoma is characterized by the loss of retinal nerve fiber tissues and the loss of the neuroretinal rim of the optic nerve head is termed glaucomatous optic neuropathy (GON). The early diagnosis of glaucoma requires measurement of intraocular pressure (IOP) by tonometry. The gold standard method widely used in clinical settings to measure IOP is Goldmann applanation tonometry (GAT). Myopia is also considered a risk factor for glaucoma. Population-based and hospital-based evidence suggests that the prevalence of GON is higher in high myopic eyes than in emmetropic eyes. The present study aims to document the association between myopia and elevated IOP.

Materials and methods

A total of 400 medium and high myopic patients attending the ophthalmology outpatient department, in a tertiary care hospital, were measured for IOP using GAT and underwent a detailed clinical evaluation. All the patients also underwent slit lamp biomicroscopic examination and fundus examination. Other data included details on demographic, socio-economic, and occupational history.

Results

A detailed refractive error examination revealed that amongst 400 right eyes, 67.5% had medium myopia, while 66.75% of the left eye were medium myopic. Amongst the medium myopic eyes, the average IOP was 15.51±3.68 mm of Hg, while in high myopic, the average IOP was 16.19±3.33 mm of Hg. A difference of 0.68 mm of Hg with high myopic eyes having higher IOP values than medium myopic eyes was observed, and it was significant statistically with a p-value <0.05.

Conclusion

There is a statistically significant association between elevated IOP and the high myopic group. In comparison to IOP measured in medium myopic and high myopic patients, elevated IOP was observed in the high myopic group than in the medium myopic group.

## Introduction

Myopia is a refractive error and is also called nearsightedness. In this condition, the rays of light coming from infinity and entering the eye parallel to the optic axis are brought to a focus in front of the retina when ocular accommodation is relaxed. This usually results from the eyeball being too long from front to back but it can also be caused by an overly curved cornea and/or a lens with increased optical power [1]. Myopia causes visual impairment in both children and adults; it is usually correctable by optical aids such as glasses and contact lenses [2]. Myopia is classified based on aetiology, clinical type, degree of myopia, and age of onset. There are four aetiological types of myopia: axial, curvatural, positional, and index myopia. Axial myopia which results from an increase in the anteroposterior length of the eyeball is considered the most common type; curvatural myopia occurs due to increased curvature of the cornea, lens, or both.

Myopia that results from an increase in the refractive index of the crystalline lens is known as index myopia and anterior placement of the crystalline lens in the eye leads to positional myopia. Clinically myopia is divided into five types: simple, degenerative, pseudo, nocturnal, and induced myopia. Simple myopia is the most common type which is generally of less than 6 D. Degenerative myopia is a higher degree of myopia with degenerative changes in the posterior segment of the eyeball. When excessive accommodation is induced due to overstimulation or ciliary spasm and by low contrast in dim illumination, it is called pseudomyopia and nocturnal myopia, respectively. Induced myopia is temporary and reversible and caused by external agents or variations in blood sugar levels. Depending upon the degree of myopia, it can be divided into three types: low myopia (<-3.0 D), medium myopia (-3.0 D to -6.0 D), and high myopia (>-6.0 D).

There are four types of myopia depending upon the age of onset: congenital, youth onset (2-20 years of age), early adult-onset (20-40 years of age), and late adult onset myopia (>40 years of age) [3]. Intraocular pressure (IOP) is determined by the production of aqueous humour into the anterior chamber by the ciliary body and its drainage via the trabecular meshwork and uveoscleral outflow from the anterior chamber. As the vitreous humour in the posterior segment has a relatively fixed volume, it does not affect IOP regulation [4]. Goldmann's equation has been a well-known reference for an adequate description of aqueous humor dynamics in clinical applications [5].

Goldmann applanation tonometry (GAT) is based on the Imbert-Fick principle, which states that within a dry thin-walled sphere, the pressure (P) inside the sphere equals the force (F) that is necessary to flatten its surface, divided by the area (A) of flattening (i.e., P = F/A) [6]. Several nomograms have been developed for adjusting GAT readings for varying central corneal thickness (CCT) [7]. Studies show that for a given IOP with primary open angle glaucoma (POAG), optic nerve damage seems to be more pronounced in highly myopic eyes with large optic discs than in emmetropic, hyperopic, or low to medium myopic eyes [8]. This association between myopia and POAG is assumed to be due to a variety of mechanisms including increased susceptibility of the optic nerve head to damage by raised IOP and the increased effect of shearing forces in optic nerve head damage. However, available evidence does not suggest a significant relationship between myopia and glaucoma in all conducted studies. Axial elongation and scleral thinning associated with myopia progression may lead to increased stress and decreased rigidity of the eyeball, thus causing an increasing trend of IOP [9]. This study aimed to assess the association between myopia and elevated IOP.

## Materials and methods

Prior consent was obtained from all participants who fulfilled the inclusion criteria and were willing to participate in the study. The study was approved by the Institutional Ethics Committee (IEC) of the Gujarat Medical Education and Research Society (GMERS), Sola.

This was a descriptive cross-sectional study conducted in a tertiary care teaching hospital in Gujarat. The sample size was calculated based on the estimated prevalence of elevated IOP as 35.6%. The estimated sample size was 353 at 95% confidence interval (CI) and a design effect of 1. Assuming a non-response rate of 10%, a total of 400 myopic patients more than 18 years of age were included in the study. It was conducted between July 2018 to July 2020. Medium or high myopic patients more than 18 years of age and willing to participate in the study were included; whereas, patients who were known cases of glaucoma, contact lens users, patients with ocular pathology (corneal diseases, inflammatory diseases, etc.), history of ocular surgery or trauma, patients with cataractous changes in the lens, and patients with astigmatism of >-3.50 D were excluded.

Data on demographic, socio-economic, and occupational history was collected. A detailed ophthalmic examination was conducted on the patients in the ophthalmology department on the same day. The parameters noted were as follows: age, gender, occupation, religion, automatic refractometer, refractive error, slit-lamp examination, IOP measurement by non-contact tonometer and GAT, measurement of central corneal thickness, and fundus examination with an indirect ophthalmoscope. CCT was measured in all patients using an ultrasonic pachymeter (DGH 550 Pachette II; DGH Technologies, Exton, PA). SPSS (IBM Corp., Armonk, NY) was used to analyse the data.

## Results

Out of a total of 400 patients included in the present study, 222 (55.5%) were female, and 178 (44.5%) were male. With a mean age of 25.20±6.57 years, patients' age ranged from 18-50 years. The maximum number of patients was between 15-25 years of age group (62.8%), followed by 26-35 years (28.7%). Out of 800 eyes screened for refractive error, it was observed that 344 (68.5%), 151 (30.1%) and seven (1.4%) eyes were from -3.0 to -5.75 D, -6.0 to -11.75 D and >-12.0 D group, respectively. Out of 800 eyes of 400 myopic patients, 537 eyes (67.1%) had refractive error between -3.0 to -5.75 D and had medium myopia. High myopic (>-6.0 D) eyes were divided into two groups of -6.0 to -11.75 D and -12.0 to -17.75 D, and there were 246 (30.7%) and 17 (2.2%) eyes. 

Table 1 describes the distribution of central corneal thickness (CCT) and the refractive error. Out of 800 eyes, 537 (67.1%) eyes had refractive error between -3.0 to -5.75 D. Amongst them, 267 (49.7%) had their CCT between 501-560 µm. There were five (29.4%) eyes from -12.0 to -17.75 D group, who had their CCT between 440-500 µm. On measuring the CCT, it was observed that almost 50% of the subjects had a CCT between 501-560, followed by 561-620 (27.62%) and 440-500 (22.12%).

**Table 1:** Distribution of Refractive Error and Central Corneal Thickness (CCT) Values Amongst the Study Population

Refractive error (D)	Central Corneal Thickness (µm)			
	440-500	501-560	561-620	Total
-3.0 to -5.75	104(19.4%)	267(49.7%)	166(30.9%)	537(67.1%)
-6.0 to -11.75	68(27.6%)	129(52.4%)	49(19.9%)	246(30.7%)
-12.0 to -17.75	5(29.4%)	6(35.3%)	6(35.3%)	17(2.2%)
Total	177(22.12)	402(50.25)	221(27.62)	800

On measuring the IOP by GAT, it was observed that out of 800, 396 (49.5%) eyes had IOP between 16-20 mm of Hg, followed by 299 (37.8%) with IOP values between 11-15 mm of Hg. Thirty-three (4.1%) had IOP between 21-25, and the rest of 11 (1.3%) eyes had their IOP >25 mm of Hg (Table 2).

**Table 2:** Distribution of Intraocular Pressure (IOP) Readings by Goldmann Applanation Tonometry Amongst Patients Included in the Study

GAT (mm of Hg)	No. of patients		
	RE	LE	Total
8-10	34(8.5%)	27(6.7%)	61(7.6%)
11-15	150(37.5%)	149(37.2%)	299(37.8%)
16-20	201(50.2%)	195(48.6%)	396(49.5%)
21-25	4(1%)	29(7.2%)	33(4.1%)
>25	11(2.7%)	0	11(1.3%)
	400	400	800 (100%)

Overall, amongst the subjects included in the present study, a maximum number of eyes had their IOP between 16-20 mm of Hg (49.5%), whereas 67.1% had refractory error between -3.0 and -5.75 D. Amongst those who had refractive error between -3.0 and -5.75 D, around 46% had normal IOP, 48% had IOP below average, and the rest 5.4% had high IOP (Table 3).

**Table 3:** Distribution of Goldmann Applanation Tonometry (GAT) Readings in Refractive Errors

Refractive error (D)	GAT (mm of Hg)					
	8-10	11-15	16-20	21-25	>25	Total (n=800)
-3.0 to -5.75 (n=537)	47 (8.7%)	211 (39.3%)	250 (46.5%)	20 (3.7%)	9 (1.7%)	537 (67.1%)
-6.0 to -11.75 (n=246)	11 (4.5%)	81 (32.9%)	141 (57.3%)	11 (4.5%)	2 (0.8%)	246 (30.7%)
12.0 to -17.75 (n=17)	3 (17.6%)	7 (41.2%)	5 (29.4%)	2 (11.8%)	0	17 (2.2%)
Total	61 (7.6%)	299 (37.37%)	396 (49.5%)	33 (4.1%)	11 (1.38%)	800

We divided myopic patients into two groups:

(1) Medium myopia (refractive error <-6.0 D)

(2) High myopia (refractive error >-6.0 D)

A detailed refractive error examination revealed that amongst 400 right eyes, 67.5% had medium myopia, while 66.75% of the left eye were medium myopic. Amongst the medium myopic eyes, the mean IOP was 15.51±3.68 mm of Hg, while in high myopic, the mean IOP was 16.19±3.33 mm of Hg. A difference of 0.68 mm of Hg with high myopic eyes having higher IOP values than medium myopic eyes was observed, and it was significant statistically with a p-value <0.05 (Table 4).

**Table 4:** Relation Between Myopia and Goldmann Applanation Tonometry (GAT)

	No. of eyes		Mean IOP±SD (mm of Hg)
	RE	LE	
Medium myopia	270 (67.5%)	267 (66.75%)	15.51±3.68
High myopia	130 (32.5%)	133 (33.25%)	16.19±3.33
Total	400	400	
Mean difference			0.68
p-value			0.0085

## Discussion

In the present study, all the patients had a refractive error of ≥-3.0 D. Out of 800 eyes, 537 (67.1%) eyes were from <-6.0 D group, and the remaining 263 (32.9%) eyes were high myopic (>-6.0 D). Mean IOP measured by GAT of medium and high myopia group was 15.51±3.68 and 16.19±3.33 mm of Hg, respectively. There was a difference of 0.68 mm of Hg between the two groups wherein high myopic eyes had numerically higher IOP measurements, which was statistically significant with a p-value <0.05.

However, in a study done by Jonas JB et al. [10] to document IOP and GON amongst 561 eyes of 261 patients, the mean age was 62±14.2 years, ranging between 13-89 years. They included high myopic patients, considering the definition of high myopia as two cut-off values of 26.5 mm and 27.5 mm of axial length. They divided it into two groups ≤27.4 mm and >27.4 mm, and studied the correlation between IOP and GON.

In a similar study by Joseph DS et al. [11] on the association between IOP and myopia in 178 eyes of 100 patients having different grades of refractive errors, the patient's mean age was 30.09 years, ranging from 11-47 years with no gender predilection. They included different grades of myopia and emmetropic patients dividing them into four groups: Group 0 - Emmetropia (+0.5 to -0.5D) included 84 (47%), Group 1 - low myopia (-0.75 to -3.00D) included 74 (42%), Group 2 -moderate myopia (-3.00 to -5.00D) included 14 (8%) and Group 3 - high myopia (>-5.00D) included six (3%) eyes. They found a significant difference in IOP between different refractive error groups. IOP in moderate and high myopic groups was high compared to emmetropic and low myopic patients, which was statistically significant.

Kumar J et al. [12] measured IOP with a Perkins applanation tonometer and the mean IOP measured in myopic patients with a refractive error of <-6.0 D and >-6.0 D (high myopia) were 13.45±2.43 and 15.40±2.18 mmHg respectively. High myopic patients having higher values of IOP with a mean difference of 1.95 mm of Hg were considered to be statistically significant.

Available evidence suggests that high myopia (in excess of −6 D) is associated with several ocular pathologies, including cataracts, retinal detachment, and glaucoma [13]. Epidemiologic evidence also suggests that moderate and high myopia with a refractive error exceeding -6D is a potential risk factor for the development and the progression of GON, with an increased risk of glaucoma compared with that of nonmyopic subjects. This risk proved to be independent of other glaucoma risk factors and IOP. Myopic eyes might have higher IOPs than emmetropic or hyperopic eyes, which may not be clinically significant [14].

The limitation of the present study is that only myopic patients were included. A comparison of IOP measurement with emmetropic and hypermetropic patients should have been done. Other variables such as glaucomatous optic disc changes and perimetry should have been taken into consideration.

## Conclusions

The present study highlighted that in comparison to IOP measured in the medium myopic group, elevated IOP was observed in the high myopic group; elevated IOP is one of the factors which initiates glaucomatous changes in the eyed, and high myopic patients are at greater risk of glaucoma. All high myopic patients should undergo glaucoma screening tests which can help in the early diagnosis of glaucoma; timely management can prevent further damage to the optic nerve.
